# A Comprehensive Evaluation of Flavor Instability of Beer (Part 1): Influence of Release of Bound State Aldehydes

**DOI:** 10.3390/foods10102432

**Published:** 2021-10-13

**Authors:** Florian Lehnhardt, Arndt Nobis, Andreas Skornia, Thomas Becker, Martina Gastl

**Affiliations:** Brewing and Beverage Technology, TUM School of Life Sciences, Technische Universität München, Weihenstephaner Steig 20, 85354 Freising, Germany; florian.lehnhardt@tum.de (F.L.); arndt.nobis@tum.de (A.N.); andreas.skornia@tum.de (A.S.); tb@tum.de (T.B.)

**Keywords:** beer aging, flavor instability, bound state aldehydes

## Abstract

Flavor instability of pale lager beer depends decisively on aroma-active aldehydes from the Maillard reaction, Strecker degradation, and lipid oxidation, which are formed in various oxidative and non-oxidative reactions. Therein, aldehydes can be formed *de novo* and be released from bound states to a free, aroma-active form during aging. During malting and brewing, proteolysis affects the amount of soluble nitrogen and thus flavor instability in different ways (e.g., precursors for *de novo* formation and binding agents for bound states). To isolate nitrogen-related aging processes, beers from malts (two barley varieties, three proteolytic malt modifications) were produced on a 50 L scale in part 1 of this study. Sensory analysis revealed increased flavor instability for beers with higher amounts of soluble nitrogen. Especially Strecker aldehydes significantly increased with malt modification. The release of bound state aldehydes revealed most free aldehydes in fresh beers and with higher malt modification. During aging, the equilibrium between free and bound state aldehydes shifted toward the free form. These results reveal a nitrogen-dependent bound pool of aldehydes that is depleted during aging and is responsible for aged aroma, especially in the early and medium stages of aging. Therefore, bound state aldehydes are indicators of the early-stage prediction of flavor instability already in a fresh condition.

## 1. Introduction

The aging of lager beer is one of the key challenges brewers face in a globalized world. During distribution and storage, reactions occur that are detrimental to the quality of beer, especially its sensory aspects [1,2].

In practice, there are various ways to assess the flavor instability of lager beer, the most common being forced aging. The sample is shaken (100 rpm, 24 h) and subjected to an elevated temperature for a certain time (40 °C, 4 day) to simulate transport and aging. After that, its aging status is evaluated using analytical and sensory techniques [3]. However, there are substantial differences in the sensory and analytical aging behaviors of forced-aged beers compared with naturally aged ones because of the elevated temperatures [4]. Because of these limitations, it is crucial to have tools to correctly describe and define the aging status of a beer sample, as well as to simulate its aging potential as early as possible (in fresh beer) [3].

The highly complex aging of pale lager beer has been thoroughly studied for several decades. It is affected by exogenous conditions (time, temperature) and endogenous parameters (pH, O_2_ concentration, pro- and anti-oxidant activity, number of precursors of aging compounds) in raw materials, during the brewing process, and in the final product [3]. Beer aging can be considered a combined multi-stage process including all these pathways and reactions [5,6].

The major contributors to aged aroma in beer are aldehydes from the Maillard reaction, Strecker degradation, and lipid oxidation, which prove to be an appropriate class of indicators for evaluating the aging status of a beer sample [7,8]. On the one hand, aldehydes can be formed *de novo* in the respective reactions or radical oxidation reactions [9]. On the other hand, aldehydes can also be released from a bound state [3,8].

*De novo* formation has been thoroughly studied, yet alone, it cannot explain the observed increases during beer aging. In a finished beer sample, reaction conditions, such as low ambient temperatures and relatively low pH (4.2–4.5), are insufficient for the initiation steps of certain reactions, such as imine formation [10].

In contrast to their free state, aldehydes can also exist in a bound state via various nucleophile additions. In beer, amino groups (amino acids), especially cysteine, and bisulfite (HSO_3_^−^) appear to be the most apparent reaction partners. The resulting imines, 2-substituted-1,3-thiazolidine-4-carboxylic acids and α-hydroxysulfonates, are hypothesized to degrade during aging and reveal the masked aldehydes [11]. In contrast, according to Bustillo Trueba et al., cysteinylated aldehydes are present in detectable concentrations in the stages from malt to wort, with a maximum at the onset of mashing but not anymore after fermentation, due to the pH instability of these compounds. Only methional (Meth) shows relatively high concentrations in a cysteinylated form [12]. The same results were obtained in worts made from malts with different proteolytic modifications. Therefore, cysteinylated aldehydes can be considered irrelevant for beer flavor instability [13]. Undoubtedly, different aldehydes show different affinities toward amino groups or HSO_3_^−^ because of their molecular structure (inductive and mesomeric effects) and are thus present in a bound state to different degrees. Up to now, this has not yet been investigated in beer.

Since arguably, bound state aldehydes are the main factors for the flavor instability of beer and, in theory, are present at the highest concentrations in fresh beer, they can be used to assess the aging potential of a fresh beer sample. For example, this can be done in the so-called nonenal potential. Hereby, the capacity of a wort to produce (E)-2-nonenal (T2N) and, since recently, also hexanal (Hex) is assessed [14,15]. Using 4-vinyl pyridine (4VP), Baert et al. developed a method to release bound state aldehydes from these bound forms through a pH shift and trapping of cysteine [16,17]. This approach can also be used to assess the aging potential of the final wort [13]. The fact that acetaldehyde (ACA) replaces other (longer-chain) aldehydes from their bound states can, in theory, also be used to predict the flavor instability of fresh beer [18,19].

All these compounds (free aldehydes, bound state aldehydes, and precursors for *de novo* formation) form the so-called aging potential of fresh beer. The totality and distribution within the aging potential vary with raw materials, especially malts, and also with the applied technology [13].

A major influence on the aging potential and thus on the formation and occurrence of aroma-active aldehydes is attributed to amino compounds. They originate mainly from the malt used in the brewing process. The number of reactants (amino compounds) in barley depends on the barley variety and crop year [20]. During germination, barley crude proteins are enzymatically degraded (proteolytic malt modification). The demanded amount of crude proteins should be in the range of 9.5–11.0% [21]. With the modification characteristics of the variety and the technological malting parameter steeping degree, the amount of soluble nitrogen and thus amino acids in the malt can be increased [22]. A practical measure of the degree of proteolysis during malting is the calculated Kolbach index, defined as the ratio of soluble protein in the laboratory wort and the total protein in the malt [21,23]. Lower amounts of soluble nitrogen and a lower Kolbach index (as long as the raw protein content is on the same level) lead to less Strecker aldehydes in the final beer [24]. Likewise, a low soluble nitrogen content provides more flavor-stable beers [25]. The specifications for the soluble nitrogen content vary in the literature. The recommendation for pale barley malt is 580–680 mg/100 g malt d.m. assessed in an ISO 65 °C mashing regime [26].

If these specifications are not met, foam stability and yeast nutrition are negatively affected. If they are exceeded, however, flavor instability increases and turbidity stability decreases [9,21]. Finally, proteolysis can be affected during the mashing procedure. Lund et al. showed that increased protease activity during mashing results in elevated levels of amino acids in final beers. During aging, these beers show significantly higher scores in the fruity aged/vinous attributes but not in papery attribute [27]. Thus, we can hypothesize that by varying reactants by proteolytic malt modification, the aging potential increases in different ways. The amount of precursor for *de novo* formation, followed by the amount of free aldehydes, and, finally, the amount of bound state aldehydes increase.

Therefore, the goal of this study was to comprehensively investigate the aging potential (*de novo* formation and release of aldehydes from bound states) that solely arises from soluble nitrogen compounds, reaction partners in both of these pathways. Furthermore, oxidative and antioxidative effects of O_2_ and SO_2_ were excluded in the final beer. Proteolytic malt modifications were used as a tool to vary the number of reactants (soluble nitrogen), as described by Nobis and Lehnhardt et al. [13]. The beers produced via a standardized brewing process without promoting further proteolysis during brewing were subjected to forced and natural aging. In part 1 of this study, we focused on (1) the sensory qualities of the respective beers, (2) the influence of soluble nitrogen on the formation of free aldehydes, and (3) the potential release of bound state aldehydes by two different methods, depending on the amount of soluble nitrogen during beer aging and their impact on the sensory qualities of the respective beers. Furthermore, we discussed the application of bound state aldehydes for the early-stage assessment of aging stability directly in fresh beer.

## 2. Materials and Methods

### 2.1. Chemicals

O-(2,3,4,5,6-pentafluorobenzyl)hydroxylamine hydrochloride (≥99%), p-fluorobenzaldehyde (98%), 2-methylpropanal (2 MP) (≥99.5%), 2-methylbutanal (2 MB) (95%), 3-methylbutanal (3 MB) (97%), 2-phenylacetaldehyde (PA) (≥90%), Meth (≥97%), benzaldehyde (Benz) (≥99.5%), pentanal (≥97.5%), Hex (98%), heptanal (95%), T2N (97%), ACA (≥99.5%), and 4-vinylpyridine (95%) were obtained from Merck (Darmstadt, Germany). 2-Furfural (Fur) (≥99.0%) was purchased from Fluka Analytical (Charlotte, NC, USA). Ethanol p.a. was purchased from VWR (Darmstadt, Germany).

### 2.2. Malt, Wort, and Beer Production

Malts (pilsner style) and worts were produced, as described by Nobis and Lehnhardt [13]. From two barley varieties, B1 (Avalon, Nordsaat Saatzucht GmbH, Langenstein, Germany) and B2 (Marthe, Saatzucht Josef Breun GmbH & Co. KG, Herzogenaurach, Germany), with a different genetically determined modification characteristic, six different malts were produced with different proteolytic modification levels by varying the steeping degree (P1: low; P2: medium; P3: high) (see Table 1).

The 6 malts were processed in a standardized scheme in duplicate, resulting in 12 worts, as previously described [13]. To avoid further proteolysis during brewing, a high-mashing-in procedure at comparable pH values and from 60 °C (mashing-in temperature) to 78 °C was used. Lautering was performed in a preheated (78 °C) lauter tun, with a lauter rest of 10 min. In total, three sparges were performed until a gravity of 10.5 °P was reached. The boiling time was 60 min. The boiled wort was transferred to a whirlpool for a 15 min rest. The cast wort (60 L, 11.5 ± 0.2 °P) was cooled to 10 °C using a plate heat exchanger.

For fermentation, dry yeast (TUM 34/70) (Fermentis, Marcq-en-Barœul, France) was rehydrated in a diluted wort (6 °P) for 6 h. The pitching rate of 15 × 106 living cells/mL was ensured using a Thoma chamber. Open fermentation was performed in cylindro-conical tanks at 15 °C (SO_2_ < 2 mg/L in beer). As the extract fell below 3.5 °P, the tanks were closed and maintained at the same temperature for 2 d. The green beers were transferred to 50 L kegs and maintained at 18 °C for 1 d. Lagering was performed at 0 °C for 4 weeks. Filtration was performed using a Seitz A20Z filter press with Seitz K150 depth filter sheets (Pall, NY, USA). After filtration, the beers were carbonated in 50 L kegs at 4 °C, filled into 500 mL bottles using a semiautomatic back pressure filler (Fillmatic, FH Maschinen und Braumanufaktur Werk II GmbH, Germany), and closed using a pneumatic corking machine (Korkfix, FH Maschinen und Braumanufaktur Werk II GmbH) to guarantee O_2_ levels below 0.1 mg/L.

### 2.3. Aging and Sample Treatment

Forced aging was performed, as previously described [4]. The bottles were shaken at 100 rpm for 24 h and then maintained at 40 °C for 4 d. Natural aging of samples was performed in a dark chamber at 20 °C until the indicated sample age was reached (1 to 9 months). At each sampling point, beer samples were filtered, aliquoted into 50 mL tubes, and immediately frozen. Samples for sensory analysis were moved to 0 °C after they reached the respective age and kept there until tasting (maximum—1 week).

### 2.4. pH, O_2_, and SO_2_

Prior to alcohol and pH analysis, beer samples were filtered. At each sampling point, the alcohol content was measured using an Anton-Paar Alcolyzer Beer ME (Graz, Austria) and the pH was measured using a pH probe. Oxygen was analyzed using an Anton-Paar CboxQC device (Graz, Austria). The bound sulfur dioxide content was determined via the destillative method (MEBAK 2.21.8.2) [28]. The latter two analyses were performed in duplicate. All other analysis was performed in triplicate.

### 2.5. Sensory Analysis

All beers were analyzed in a fully randomized setup in a single repetition by, on average, 10 (ranging from 9 to 13) panelists trained and certified by Deutsche Landwirtschafts–Gesellschaft e.V. (DLG). The panelists underwent continuous training (once per week). Three different (two rating and one descriptive) sensory methods were used. In each session, aging-relevant sniffing samples were provided as an introductory exercise and a commercial fresh pale lager beer (not older than 4 weeks) was presented as a control sample. The samples were served in brown glasses at 10 °C ± 2 °C.

First, quality assessment was performed according to the DLG 5-point scheme. Five categories (purity of smell, purity of taste, palate fullness, freshness, and quality of bitterness) were rated on the following monadic scale: 0 = inadequate (not evaluable); 1 = not satisfactory (strong deviation); 2 = less satisfactory (clear deviation); 3 = satisfactory (perceptible deviation); 4 = good (slight deviation); and 5 = very good (quality expectations reached in full). A weighted overall DLG score was calculated as follows:

Weighted overall DLG score = [(2 × purity of smell) + (2 × purity of taste + palate fullness + freshness) + (2 × quality of bitterness)]/8

Second, the aging-specific quality scheme according to Eichhorn was used. Three categories (smell, taste, and bitterness) were rated on the following monadic scale with the possibility of 0.5 steps: 1 = fresh beer, no aging impressions; 2 = slight aging impressions; 3 = strong aging impressions, acceptancy threshold for consumers; and 4 = extreme aging impressions, such as sherry. Only aging-relevant impressions were judged. Acceptance according to the Eichhorn scheme was evaluated on a hedonic scale from 0% (no acceptance) to 100% (full acceptance).

Finally, check all that apply (CATA) was used to describe aging-relevant aromas. The attributes fruity, berry, sweetish, honey, cardboard, bready, sherry, and cooked vegetables were checked if present and not checked if absent. Panelists were also able to provide free comments. The data were obtained as the sum of checks per attribute by the number of panelists.

To evaluate whether the aging character of the produced beers differs between the malt modifications, triangle tests with 16 tasters for all combinations per barley variety at 9 months (M9) were performed according to MEBAK Sensorik 3.1.3 [29]. Therefore, beers from the repeated brews were blended. The samples were presented in a fully randomized setup.

### 2.6. Quantitation of Free Aldehydes via HS-SPME-GC-MS

Headspace–solid phase microextraction–gas chromatography–mass spectrometry (HS-SPME-GC-MS) was carried out according to Lehnhardt et al. with minor modifications [4]. A cooled beer sample (5 mL) was transferred together with 50 µL of an internal standard (2 mg/L of p-fluorobenzaldehyde in ethanol) to a 20 mL headspace vial and stored in a cooled autosampler tray (17 °C). Extraction was performed using a CAR-PDMS-DVB fiber. First, the fiber was loaded with o-(2,3,4,5,6-pentafluorobenzyl)hydroxylamine for 10 min at 40 °C. Then, the headspace of the sample was extracted for 30 min at 40 ℃. The fiber was injected with a 1/5 split at 270 °C into a gas chromatography (GC) instrument (GC-Ultra 1300; Thermo Fisher Scientific, Waltham, MA, USA) coupled to a single quad mass spectrometer (ISQ 7000; Thermo Fisher Scientific). The GC instrument was equipped with a DB-5 column (length, 60 m; inner diameter, 0.25 mm; film thickness, 0.25 µm; Thermo Fisher Scientific). The carrier gas was helium (flow rate 1.85 mL/min). The starting temperature was maintained at 60 °C for 4 min, followed by heating at 5 K/min up to a final temperature of 250 °C, which was maintained for 3 min. A full scan mode (*m*/*z* 35–350) with a dwell time of 0.02 s was applied for the analysis. Each sample was analyzed in triplicate. Peak detection was performed using Xcalibur 3.1.66.10 (Thermo Fisher Scientific). Quantification was achieved by external calibration. The lowest calibration point was defined as the limit of quantification (LOQ), which was in accordance with previous studies [30,31]. All measurements were performed in biological duplicate and technical triplicate.

### 2.7. Quantitation of Bound State Aldehydes after Release with 4VP vs. Acetaldehyde (ACA)

The procedure was performed as described in Section 2.6, with one exception. Before adding the internal standard, 50 µL of 4VP solution (1/1 4VP/ethanol, *v*/*v*) was added or in the case of competitive release with ACA, 50 µL of ACA stock solution (50 mg/mL) was added. The samples were incubated in an autosampler tray at 17 °C for at least 6 h in the case of 4VP and 12 h in the case of ACA. During 4VP elution from the GC column, mass spectrometric detection was turned off.

The degree of bound state aldehydes was calculated as follows:

c(bound) (%) = (c(released) − c(free))/(c(free)),

where c(released) and c(free) are defined as concentrations of released versus free aldehydes.

### 2.8. Statistical Analysis

Data analysis was performed using JMP Pro 14 (SAS Institute Inc., Cary, NC, USA). From technical and biological multiplicates, means and standard deviations were calculated. One-way analysis of variance (ANOVA) was performed to determine statistical differences, where indicated. Post hoc testing for the comparison of all pairs was achieved with the Tukey–Kramer honestly significant difference (HSD) test. Unless stated otherwise, α = 0.05 was used and each analysis was performed in technical triplicate.

## 3. Results and Discussion

### 3.1. Brewing Trials

To comprehensively investigate all aspects of the aging potential, beers were produced from two different barley varieties (B1: Avalon; B2: Marthe) with variations in the proteolytic malt modification level (P1: 550 mg/100 g malt d.m.; P2: 625 mg/100 g malt d.m.; P3: 700 mg/100 g malt d.m.). Table 1 shows these malt specifications as well as pH, O_2_, and bound SO_2_ amounts of the fresh beers analyzed directly after filling.

Proteolytic malt specifications were achieved through different steeping degrees during malting and goals set to the extrema of specifications, including a medium amount to obtain detectable but still realistic differences. The targets of the individual modification measured as soluble N were satisfactorily reached for all variations. The total amount of amino acids increased with proteolytic malt modification, thus providing more reactants and precursors for aging-relevant reactions. The only exception was B1/P2. During proteolysis, all amino acids increased except for proline. This decrease led to a lower amount of total amino acids in B1/P2 (Table S1).

In malt, the pH values were similar (Table S1). The fresh beers, in contrast, showed significant differences in pH within the acceptable range for pale lager beers. P2 showed the lowest pH in both barley varieties, most likely due to the optimal nutritional value for yeast provided in the wort and the resulting better pH drop during fermentation.

The O_2_ levels directly after filling were minimal (<0.1 mg/L) in all samples, as reported earlier [10]. The bound SO_2_ target was set to <1 mg/L and was reached for all but one sample (B2/P3 varied from 1.9 to 4.3 mg/L in duplicate beers). However, the bound SO_2_ concentration was still low and thus acceptable. The minimal O_2_ and SO_2_ concentrations were optimal to isolate N-related aging processes. On the one hand, direct oxidation through reactive oxygen species formation is limited, and on the other hand, antioxidative effects and covalent binding of aldehydes are excluded. Therefore, the beers produced in this study were ideal for the isolated investigation of the aging potential provided by N species and were subjected to forced (FO) and natural aging of up to 9 months (M1–M9). Together with fresh samples (FR), all aging points were tasted using a trained sensory panel and analyzed by instrumental analytics, as described next.

### 3.2. Sensory Analysis

An important part of the evaluation of flavor instability is sensory analysis with ratings (descriptive and discriminative methods). This way, changes in product quality during aging can be assessed and differences in the aging potential unraveled.

#### 3.2.1. Quality Assessment and Descriptive Analysis by DLG, Eichhorn Scheme, and CATA

First, the sensory quality of the produced beers was investigated during aging. Differences in sensory analysis after a certain period of aging indicate an influence of the proteolytic malt modification.

Aging clearly influenced the analyzed samples. A continuous increase of aging impression was observed after M3 in several aspects. The rating in different groups suggested sensory distinguishability. For example, the M1 sample showed the highest DLG overall scores, followed by FR, FO, M2, M3, M4, M5, M6, and M9 samples. Interestingly, fresh and forced-aged samples did not show differences in these attributes. For smell, according to the Eichhorn scheme, the same results were obtained. The M9 sample scored the highest in this attribute, followed by M6, M5, M4, M3, M2, FR, FO, and M1 samples. Hedonic acceptance was the highest for the M1 sample and decreased in FR, FO, M2, M3, M4, M6, M5, and M9 samples. Furthermore, M3–M9 samples showed elevated scores in the attribute bready: M6, M4, M9, M5, and M3 samples in contrast to M1, FR, M2, and FO samples. The older samples, M6 and M9, showed elevated scores in the attribute honey and the M9 sample also showed elevated scores in the attribute sherry. These results indicate that aging has a perceivable effect on beer quality after M3 (see Table S2).

**Figure 1:**
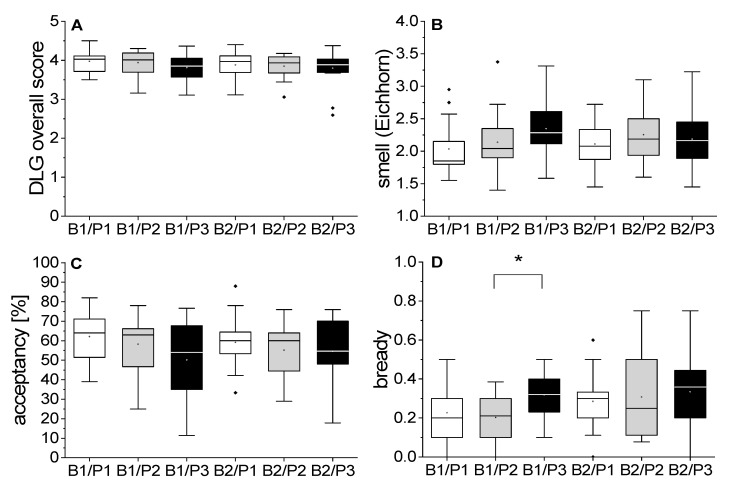
Sensory analysis of beers: (**A**) DLG overall score, (**B**) smell according to the Eichhorn scheme, (**C**) acceptancy (%), and (**D**) attribute bready. Boxplots show values of all aging points (*n =* 18). Linked boxplots showed a significant difference (*: *p* < 0.05). (B1: barley variety 1; B2: barley variety 2; P1: low proteolytic malt modification; P2: medium proteolytic malt modification; P3: high proteolytic malt modification).

Furthermore, we investigated the influence of proteolytic malt modification. Figure 1 shows boxplots of chosen attributes of standardized brewed beers by barley variety and malt modification level. One boxplot includes all evaluated aging points (fresh, forced aged, and naturally aged (M1–M9)).

In addition, the malt modification level influenced the aging behavior of the analyzed beer samples. For B1, we observed a stronger aging impression (lower DLG scores, higher Eichhorn scores, higher intensities for aging descriptors) and reduced acceptance with a higher amount of soluble N. Although the results showed no statistical significance, we found strong trends (one-way ANOVA at α = 0.05; *p* = 0.21 (Figure 1A), 0.11 (Figure 1B), 0.07 (Figure 1C), 0.02 (Figure 1D)). The only exception was observed for the attribute bready. The B1/P2 and B1/P3 pair showed significant differences (Tukey–Kramer HSD test, *p* = 0.02). For B2, the same trends were observed but generally on a weaker level (one-way ANOVA at α = 0.05; *p* = 0.79 (Figure 1A), 0.59 (Figure 1B), 0.60 (Figure 1C), 0.72 (Figure 1D).

These analyses revealed no significant differences but only trends between proteolytic malt modification when all sampling dates were considered together. Still, this does not imply that the aging potential of individual beer samples does not differ between proteolytic malt modification.

#### 3.2.2. Triangle Tests

Second, the goal was to determine whether the beers showed differences in a discriminative test (triangle test according to MEBAK Sensorik 3.1.3) after a long-term natural aging period (M9), as suggested by the aforementioned sensory results. M9 was chosen because the oldest sample in this study could unfold its aging potential to the highest degree. Table 2 shows the results of the triangle test. *p*-Values in bold indicate statistically significant results (α = 0.05).

After M9, the triangle test did not reveal significant differences for B1/P1 and B1/P2, because these samples showed the smallest difference in soluble N (28 mg/100 g malt d.m.) (Table 1). However, for all other pairs, significant differences were observed. Especially, all pairs with P3, samples with the highest amounts of soluble N, showed clear results. This was the case even for B2/P3, although this sample showed the highest amount of bound SO_2_ in fresh beers (Table 1). The values ranged from 1.9 to 4.4 mg/L in duplicate brews, indicating that little to medium amounts of SO_2_ have no positive effect on flavor instability after M9, independent of the amount of soluble N.

Therefore, sensory analysis of the produced beers revealed differences in the aging behavior that depended on the amount of soluble N. With increasing proteolytic malt modification, we observed decreased sensory beer quality and acceptance. After 9 months of natural aging, almost all pairs showed significant differences in the triangle test, indicating a varied aging potential in fresh beer.

### 3.3. Behavior of Free Aldehydes

The clear sensory differences, especially for the attribute bready, in the produced samples during aging indicated that the amount of soluble N affects the number of aroma-active compounds. Amino acids, as part of the soluble N in beer, are reactants in various aldehyde-yielding reactions and direct precursors of Strecker aldehydes. These aldehydes are used as aging indicators because of the fact that they increase during aging but show high aroma activity [4]. Next, we discuss the behavior of free aldehydes: 2MP, 2MB, 3MB, Meth, PA, Benz, Fur, Hex, and T2N.

**Figure 2:**
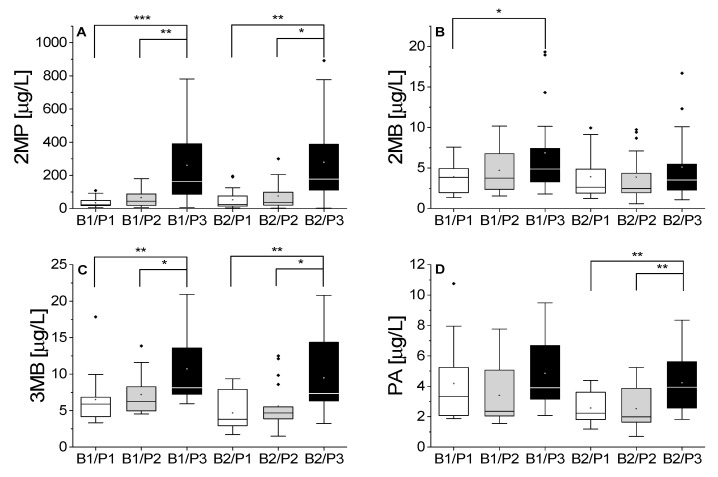
Boxplots of all aging points of chosen aldehydes ((**A**) 2MP; (**B**) 2MB; (**C**) 3MB; (**D**) PA) by malt modification level (B1: barley variety 1; B2: barley variety 2; P1: low proteolytic malt modification; P2: medium proteolytic malt modification; P3: high proteolytic malt modification). Linked boxplots showed a significant difference (*: *p* < 0.05; **: *p* < 0.01; ***: *p* < 0.001).

Figure 2 shows the concentrations of chosen aldehydes that showed statistically significant differences. Each boxplot contains the data of all aging points (FR, FO, M1–M9). The behavior of all other investigated aldehydes can be found in Table S3.

In the fresh condition, we observed none to only minor differences in the analyzed aldehydes between different malt modification levels (Table S3 and part 2). Generally, all aldehydes increased during aging to different extents, depending on the amount of soluble N in the samples. This indicated either N-dependent *de novo* formation or a release from the bound state. Especially, the concentration of Strecker aldehydes (2MP, 2MB, 3MB, and PA) in beer significantly increased at higher malt modification levels. Again, higher proteolytic modification (P3) showed a significant increase in the content of these aldehydes. Meth was mostly present only below its LOQ, and thus the results showed no dependency on the malt modification level despite Meth being a Strecker aldehyde. Other aldehydes such as Fur, Benz, Hex, and T2N were not significantly influenced by the amount of soluble N. Fur is mostly influenced by the heat load during beer production [8]. Benz, even though being considered a Strecker aldehyde, appears to be more dependent on oxygen [6]. The formation of Hex and T2N is influenced by the concentration of lipids (varying by environment and cultivar) and the enzymes involved in their degradation during malting and brewing. Thus, their concentration is independent of the N species [20].

The observed increases in Strecker aldehydes explain the sensory impressions of aged beers, since these compounds are important contributors to aged aroma and especially to bready impressions [32]. A higher proteolytic malt modification lead to a higher aging potential in fresh beer and thus to increased formation of aldehydes during aging. An increased number of reactants during beer production results in a higher aging potential in the wort, which is likely to be transferred to the final beer [13].

### 3.4. Release of Bound State Aldehydes

The aging potential comes from reactive precursors, free aldehydes, and bound state aldehydes. These bound state aldehydes are relevant sources, next to *de novo* formation, of free aldehydes [3]. Thus, the behavior of bound state aldehydes during beer aging was assessed by two different methods.

First, bound state aldehydes can be released by the addition of 4VP prior to analysis. The released free aldehydes can be determined by HS-SPME. 4VP is a cysteine-trapping reagent and shows high reactivity toward thiols; thus, it acts as a binding reagent toward nucleophiles. Furthermore, the addition of 4VP to beer samples leads to a pH shift into the weak alkaline state. Baert et al. found high recoveries in model systems [11]. They ignored the observed release of bound state aldehydes from *de novo* formation of free aldehydes, observed the release of bound state aldehydes, and found a remarkable variation in release-able aldehydes between different beer samples [17].

Second, bound state aldehydes can be released by excessive addition of ACA, which acts as a competitive agent toward other aldehydes that occur in a bound state. Upon addition, the chemical equilibrium changes and ACA being the most electrophile aldehyde subsequently pushes out other aldehydes into their free form. Thus, these compounds can be determined as free aldehydes by HS-SPME [18].

#### 3.4.1. Release by 4VP

**Figure 3:**
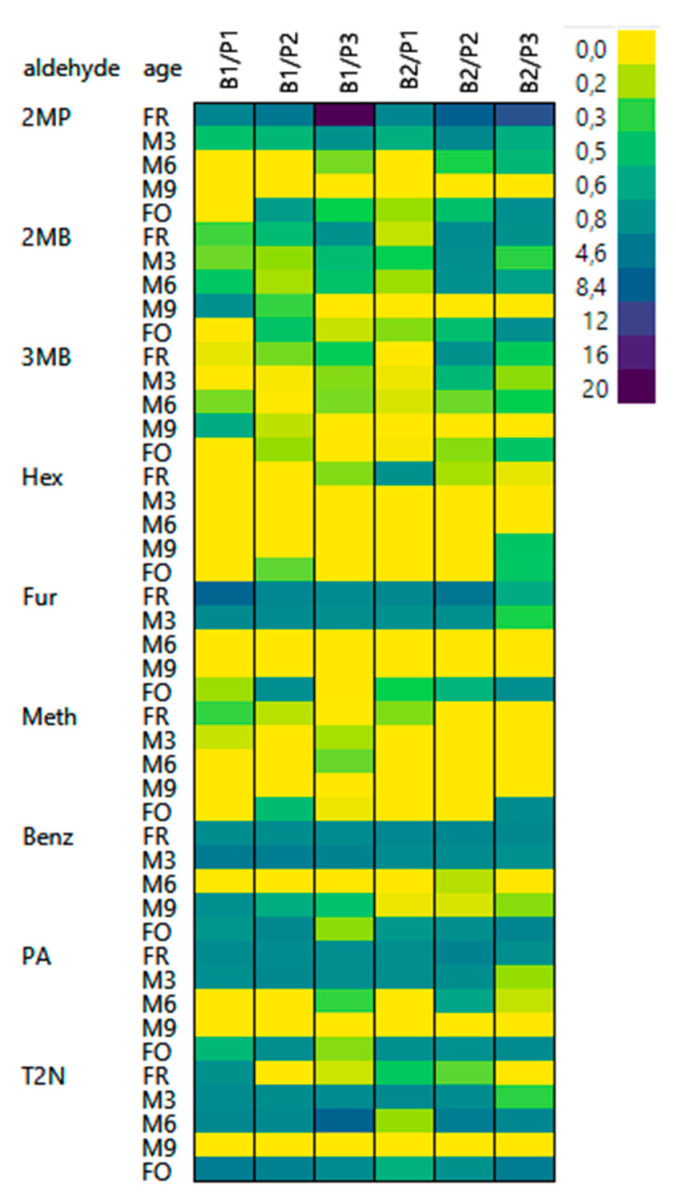
4VP-releasable aldehydes (*n* = 6): the heatmap shows the relative concentration of bound state aldehydes in comparison to the free form (B1: barley variety 1; B2: barley variety 2; P1: low proteolytic malt modification; P2: medium proteolytic malt modification; P3: high proteolytic malt modification).

Figure 3 shows the degree of bound state aldehydes after release by 4VP. The degree is a relative variable calculated as described before. A value of 0 indicates no release of bound state aldehydes, while values > 0 imply release.

The degree of bound state aldehydes in the analyzed beers varied by the status of aging, malt modification level, and type of aldehyde. In most fresh samples, considerable amounts of aldehydes could be released by 4VP. The degree of releasable aldehydes decreased during aging. After M9, no more aldehydes could be released in most cases. This over-time-decreasing amount of bound state aldehydes was statistically significant for 2MP (one-way ANOVA: *p* = 0.0002), Fur (one-way ANOVA: *p* = 0.0004), and PA (one-way ANOVA: *p* < 0.0001). These compounds appeared to be the most promising aldehydes for use as early-stage indicators of flavor instability in fresh beer. Furthermore, Benz (one-way ANOVA: *p* < 0.0001) and T2N (one-way ANOVA: *p* = 0.0024) showed significant differences during aging. However, for these compounds, most aldehydes could be released after M3 versus M6 and forced aging. Apparently, bound state aldehydes are also released during forced aging, although to varying extents. In the case of T2N, more aldehydes were releasable after forced aging.

#### 3.4.2. Release by ACA

**Figure 4:**
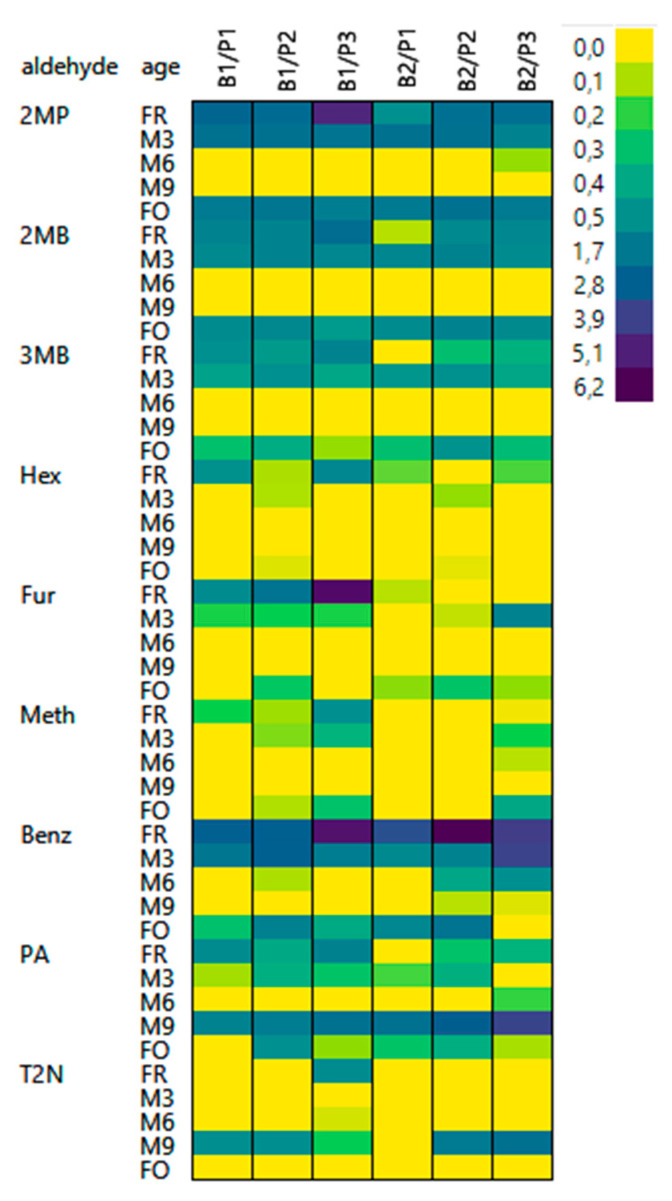
ACA-releasable aldehydes (*n* = 6): heatmap shows relative concentration of bound state aldehydes in comparison to the free form (B1: barley variety 1; B2: barley variety 2; P1: low proteolytic malt modification; P2: medium proteolytic malt modification; P3: high proteolytic malt modification).

Figure 4 shows the degree of bound state aldehydes after release by ACA.

As observed before, the degree of bound state aldehydes released by ACA varied by the status of aging, malt modification level, and type of aldehyde. For most samples, the number of ACA-releasable aldehydes showed a decrease during aging. More aldehydes could be released from fresh samples compared to natural and forced-aged samples. This was especially true for 2MP (one-way ANOVA: *p* < 0.0001), 2MB (one-way ANOVA: *p* < 0.0001), 3MB (one-way ANOVA: *p* < 0.0001), Hex (one-way ANOVA: *p* = 00073), and Benz (one-way ANOVA: *p* ≤ 0.0001). Fur (one-way ANOVA: *p* = 0.12) and Meth (one-way ANOVA: *p* = 0.24) showed the same behavior but not significantly. PA and T2N also showed a similar behavior, with the exception that the highest concentrations of these compounds could be released at M9.

### 3.5. Influence of Aldehyde Structure on Occurrence in a Bound State

The chemical structure of aldehydes influences the degree of binding in various ways. Generally, steric effects might hinder the binding of Fur, Benz, and PA. Positive inductive effects (+I) lower electrophilicity and thus the binding affinity (e.g., for 2MB compared with 3MB). Positive mesomeric effects (+M) hinder the binding of Fur, Benz, and PA due to a higher electron density at the carbonyl group [33]. Bueno et al. investigated the equilibrium constants (K_a_) of a variety of aldehydes and HSO_3_^−^ in model wines. Higher values indicated that the equilibrium is more on the side of the bound state aldehyde. They found that along with ACA (K_a_ = 485 × 10^3^), Meth (K_a_ = 50 × 10^3^) and 3MB (K_a_ = 29 × 10^3^) showed higher affinity toward HSO_3_^−^ compared to PA (K_a_ = 17 × 10^3^), 2MP (K_a_ = 2.8 × 10^3^), 2MB (K_a_ = 2.6 × 10^3^), or Fur (K_a_ = 0.1 × 10^3^). Higher values indicate an increased affinity toward nucleophiles [14,15].

In fresh beers, 2MP (up to 1970%), Fur (up to 760%), and PA (up to 290%) showed the highest relative release rates in the case of 4VP and, at the same time, together with 3MB, the highest absolute concentrations (Table S3). Thus, not only Ka but also process technology needs to be considered. Although 2MP is the most volatile of these compounds, it was also present in the free form in the highest quantities and might withstand processes such as evaporation during boiling at relatively high concentrations compared to other compounds. Fur and PA, in contrast, have relatively low volatilities and thus are not evaporated to the same extent but rather remain in the liquid phase. Therefore, these compounds might react more with nucleophiles in the matrix and thus occur more in a bound state. The results obtained in this study are in agreement with the literature. Baert et al. found that T2N, Fur, and 2MP could be released from fresh commercial beers by 4VP, each at an increase of more than 100% [17].

ACA revealed the presence of 2MP (up to 490%), 2MB (up to 220%), 3MB (up to 120%), Hex (up to 102%), and Benz (up to 620%) in bound states in fresh beers and their depletion during aging. Fur (except for B1/P3) and Meth did so, too, but to lesser extents. Interestingly, PA and T2N showed the same behavior, with the exception that after M9, more aldehydes were again released upon the addition of ACA. The highest absolute concentrations in the bound state were obtained for 2MP, 3MB, and PA (Table S3). The same aldehydes were among the ones that could be released by 4VP at high concentrations.

### 3.6. Final Discussion of Release and Bound State Aldehydes

**Figure 5:**
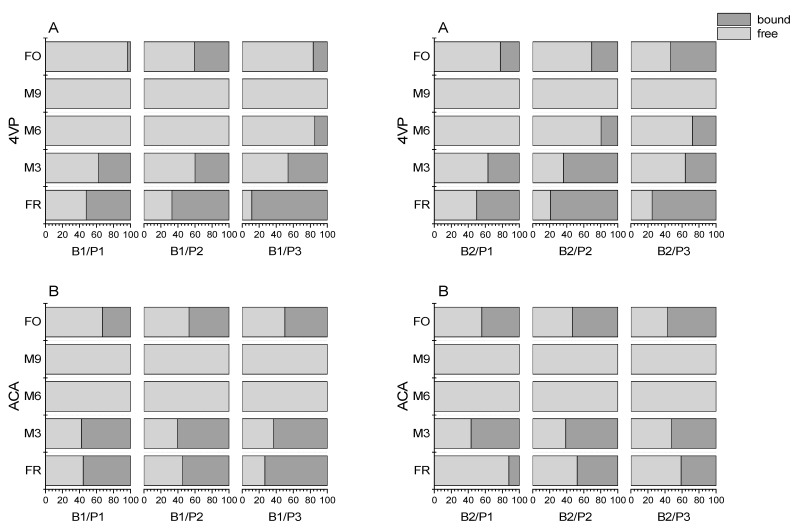
Ratio of sum of free and bound state aldehydes assessed by (**A**) 4VP and (**B**) ACA (B1: barley variety 1; B2: barley variety 2; P1: low proteolytic malt modification; P2: medium proteolytic malt modification; P3: high proteolytic malt modification).

Based on both these release methods, we finally investigated whether all the aging-related aldehydes are released during aging. Therefore, the sums of all investigated free and bound state aldehydes were calculated. Figure 5 shows the equilibrium between the sum of free aldehydes and the sum of bound state aldehydes assessed by the two applied release methods. We observed that the equilibrium shifted toward the free form during aging. After M9 versus M6, the equilibrium was fully toward the free form, indicating either hydrolysis or a different degradation of an important bound state fraction. Further possibilities could be the irreversible binding on proteins [10], proline-catalyzed aldol condensation [34], or further oxidation reactions, which will be discussed in part 2 of this study.

Furthermore, the equilibrium was influenced by the amount of soluble N in the sample. In fresh samples, the ratio of bound state and free aldehydes increased with the amount of soluble N for 4VP, and ~82% of aldehydes were free in P3, ~73% in P2, and 51% in P1 (R = 0.69). The same result was observed for ACA but to a lower extent (~57% in P3, 51% in P2, and 34% in P1; R = 0.26). A higher proteolytic malt modification level resulted in elevated concentrations of all amino acids except proline (see part 2). Therefore, amino acids are important binding agents for aldehydes. It was assumed that cysteinylated aldehydes have a major influence on beer flavor instability. In fact, they do exist in the beer matrix but only at negligible concentrations and increase during aging since they are in equilibrium with free aldehydes [12]. Thus, the N adducts that we observed in this study are more likely to be in the form of imines, either with amino acids or with larger peptides and proteins. These compounds would be hydrolyzed slowly during beer aging and thus would release free aldehydes. The degradation is favored at pH 4.0 because of the presence of a zwitterionic hemiaminal. Therefore, the targeted analysis of imines after reduction, as described in the literature, is necessary in future research [35]. Furthermore, untargeted analysis of protein-bound aldehydes seems highly promising for further elucidation of bound state species.

In summary, 4VP can release more bound state aldehydes compared with ACA. 4VP acts via a competitive (for nucleophiles) mechanism and also elevates the pH of the sample. ACA only acts competitively. Using ACA is the softer, less invasive way of releasing bound state aldehydes. Ultimately, beer is a dynamic equilibrium system, and each method can only shift the equilibrium to a certain extent. Both presented methods, 4VP- and ACA-induced release, are promising tools for the early-stage assessment of beer flavor instability regardless of the maximum degree of release. Which method comes closest to reality is highly influenced by the samples, its way of production, and, ultimately, storage conditions.

Contrary to the literature, 4VP does not mostly release SO_2_ adducts, as observed in beers with up to 10 mg/L of SO_2_ [17]. 4VP and ACA can also release N-related adducts.

## 4. Conclusions

Samples brewed via a standardized brewing process with a high-mashing-in procedure from two different barley varieties at different proteolytic malt modification levels showed different aging behaviors in sensory analysis, as well as free and bound state aldehydes. The soluble N in these samples ranged from 569 to 731 mg/100 g malt d.m. (ISO 65 °C mashing procedure), covering both low and high limits of the demanded brewing specifications. The sensory and analytical aging status increased with the amount of soluble N. The increase in free aldehydes, especially in the early to medium stage of aging (up to M6 of natural aging) can be explained by the release of bound state aldehydes to a strong degree. The impact of *de novo* formation will be discussed in part 2 of this study.

Aldehydes have different affinities with regard to their form of occurrence (free or bound) due to their chemical structures. The degree of binding is a combined result of abundance of the aldehyde and its electrophilicity. This equilibrium of free and bound state aldehydes shifts toward the free form during aging. After M9 versus M6, depending on the release method, none or only a negligible impact of bound state aldehydes on the flavor instability of beer was observed. Therefore, the assessment of bound state aldehydes after their release is a promising alternative analytical method of forced aging and allows for early-stage prediction of the aging potential without thermal intake and the related problems in fresh beer.

## Figures and Tables

**Table 1:** Malt and brewing specifications: steeping degree, soluble N (target and real), total amino acids, pH, O_2_, and bound SO_2_.

Sample	Steeping Degree (%)	Target Soluble N (ISO 65 °C (mg/100 g Malt d.m.)	Soluble N (mg/100 g Malt d.m.)	Total Amino Acids in Fresh Beer (mg/L)	pH (Final Beer)	O_2_ (mg/L)	Bound SO_2_ (mg/L)
B1/P1	38	550 ± 25	573 ± 10	909	4.57 ± 0.02	0.07 ± 0.04	0.84 ± 0.33
B1/P2	41	625 ± 25	601 ± 1	753	4.44 ± 0.05	0.01 ± 0.00	0.52 ± 0.20
B1/P3	44	700 ± 25	660 ± 1	980	4.52 ± 0.07	0.02 ± 0.00	0 ± 0
B2/P1	39	550 ± 25	569 ± 3	666	4.45 ± 0.07	0.07 ± 0.05	0 ± 0
B2/P2	43	625 ± 25	620 ± 14	784	4.39 ± 0.05	0.08 ± 0.01	0.16 ± 0.18
B2/P3	47	700 ± 25	731 ± 1	1121	4.55 ± 0.02	0.02 ± 0.01	3.16 ± 1.43

**Table 2:** Triangle tests to determine sensory differences in beer after 9 months of natural aging (*n* = 16) (B1: barley variety 1; B2: barley variety 2; P1: low proteolytic malt modification; P2: medium proteolytic malt modification; P3: high proteolytic malt modification).

Tested Pair	Correct Answers	Wrong Answers	*p*-Value
B1/P1–B1/P2	8	8	>0.05
B1/P1–B1/P3	12	4	<0.001
B1/P2–B1/P3	13	3	<0.001
B2/P1–B2/P2	10	6	<0.01
B2/P1–B2/P3	14	2	<0.001
B2/P2–B2/P3	14	2	<0.001

## Data Availability

The data presented in this study are available within the article.

## Supplementary Materials

The following are available online at https://www.mdpi.com/article/10.3390/foods10102432/s1, Table S1: malt and beer analysis, Table S2: sensory results, Table S3: free and bound aldehydes.
